# Supplementary material for: Discovery and application of insertion-deletion (INDEL) polymorphisms for QTL mapping of early life-history traits in Atlantic salmon
Source: BMC Genomics. 2010 Mar 8;11:156. doi: 10.1186/1471-2164-11-156 (PMC2838853; doi:10.1186/1471-2164-11-156)
Supplement: Additional file 2 — Information on developed 76 locus single-run INDEL panel in Atlantic salmon. Information on fluorescence labeling, primer concentrations, PCR pooling and links to alignments, INDEL motifs and GENESCAN (Burge and Karlin 1997) predictions of genes/exons are available in html format. [file 1471-2164-11-156-S2.ZIP › Additionalfile2/table13066.html]

Contig Alignment

```
Base	A B C D E F G H I J K L   
1	. G . . . . . . . . . .    
2	. G . . . . . . . . . .    
3	. G . . . . . . . . . .    
4	. G . . . . . . . . . .    
5	. A . . . . . . . . . .    
6	. T . . . . . . . . . .    
7	. G . . . . . . . . . .    
8	. T . . . . . . . . . .    
9	. T . . . . . . . . . .    
10	. C . . . . . . . . . .    
11	. C . . . . . . . . . .    
12	. T . . . . . . . . . .    
13	. G . . . . . . . . . .    
14	. G . . . . . . . . . .    
15	. A . . . . . . . . . .    
16	. C . . . . . . . . . .    
17	. A . . . . . . . . . .    
18	. T . . . . . . . . . .    
19	. C . . . . . . . . . .    
20	. T . . . . . . . . . .    
21	. A . . . . . . . . . .    
22	. C . . . . . . . . . .    
23	. A . . . . . . . . . .    
24	. A . . . . . . . . . .    
25	. C . . . . . . . . . .    
26	. T . . . . . . . . . .    
27	. C . . . . . . . . . .    
28	. C . . . . . . . . . .    
29	. T . . . . . . . . . .    
30	. A . . . . . . . . . .    
31	. C . . . . . . . . . .    
32	. C . . . . . . . . . .    
33	. A . . . . . . . . . .    
34	. A . . . . . . . . . .    
35	. C . . . . . . . . . .    
36	. A . . . . . . . . . .    
37	. G . . . . . . . . . .    
38	. C . . . . . . . . . .    
39	. G . . . . . . . . . .    
40	. G . . . . . . . . . .    
41	. C . . . . . . . . . .    
42	. G . . . . . . . . . .    
43	. G . . . . . . . . . .    
44	. C . . . . . . . . . .    
45	. T . . . . . . . . . .    
46	. G . . . . . . . . . .    
47	. G . . . . . . . . . .    
48	. A . . . . . . . . . .    
49	. G . . . . . . . . . .    
50	. A . . . . . . . . . .    
51	. A . . . . . . . . . .    
52	. G . . . . . . . . . .    
53	. G . . . . . . . . . .    
54	. A . . . . . . . . . .    
55	. G . . . . . . . . . .    
56	. C . . . . . . . . . .    
57	. A . . . . . . . . . .    
58	. G . . . . . . . . . .    
59	. G . . . . . . . . . .    
60	. C . . . . . . . . . .    
61	. T . . . . . . . . . .    
62	. G . . . . . . . . . .    
63	. C . . . . . . . . . .    
64	. C . . . . . . . . . .    
65	. C . . . . . . . . . .    
66	. A . . . . . . . . . .    
67	. A . . . . . . . . . .    
68	. G . . . . . . . . . .    
69	. T . . . . . . . . . .    
70	. C . . . . . . . . . .    
71	. C . . . . . . . . . .    
72	. A . . . . . . . . . .    
73	. G . . . . . . . . . .    
74	. G . . . . . . . . . .    
75	. C . . . . . . . . . .    
76	. C . . . . . . . . . .    
77	. T . . . . . . . . . .    
78	. A . . . . . . . . . .    
79	. G . . . . . . . . . .    
80	. A . . . . . . . . . .    
81	. T . . . . . . . . . .    
82	. G . . . . . . . . . .    
83	. G . . . . . . . . . .    
84	. G . . . . . . . . . .    
85	. G . . . . . . . . . .    
86	. T . . . . . . . . . .    
87	. C . . . . . . . . . .    
88	. A . . . . . . . . . .    
89	. A . . . . . . . . . .    
90	. A . . . . . . . . . .    
91	. G . . . . . . . . . .    
92	. G . . . . . . . . . .    
93	. T . . . . . . . . . .    
94	. C . . . . . . . . . .    
95	. A . . . . . . . . . .    
96	. T . . . . . . . . . .    
97	. T . . . . . . . . . .    
98	. A . . . . . . . . . .    
99	. C . . . . . . . . . .    
100	. T . . . . . . . . . .    
101	. A . . . . . . . . . .    
102	. G . . . . . . . . . .    
103	. T . . . . . . . . . .    
104	. A . . . . . . . . . .    
105	. G . . . . . . . . . .    
106	. T . . . . . . . . . .    
107	. C . . . . . . . . . .    
108	. A . . . . . . . . . .    
109	. C . . . . . . . . . .    
110	. C . . . . . . . . . .    
111	. C . . . . . . . . . .    
112	. T . . . . . . . . . .    
113	. C . . . . . . . . . .    
114	. T . . . . . . . . . .    
115	. G . . . . . . . . . .    
116	. A . . . . . . . . . .    
117	. C . . . . . . . . . .    
118	. A . . . . . . . . . .    
119	. G . . . . . . . . . .    
120	. C . . . . . . . . . .    
121	. A . . . . . . . . . .    
122	. C . . . . . . . . . .    
123	. C . . . . . . . . . .    
124	. C . . . . . . . . . .    
125	. T . . . . . . . . . .    
126	. A . . . . . . . . . .    
127	. G . . . . . . . . . .    
128	. T . . . . . . . . . .    
129	. G . . . . . . . . . .    
130	. G . . . . . . . . . .    
131	. G . . . . . . . . . .    
132	. C . . . . . . . . . .    
133	. A . . . . . . . . . .    
134	. G . . . . . . . . . .    
135	. A . . . . . . . . . .    
136	. A . . . . . . . . . .    
137	. C . . . . . . . . . .    
138	. T . . . . . . . . . .    
139	. C . . . . . . . . . .    
140	. C . . . . . . . . . .    
141	. C . . . . . . . . . .    
142	. A . . . . . . . . . .    
143	. T . . . . . . . . . .    
144	. G . . . . . . . . . .    
145	. G . . . . . . . . . .    
146	. C . . . . . . . . . .    
147	. T . . . . . . . . . .    
148	. G . . . . . . . . . .    
149	. T . . . . . . . . . .    
150	. G . . . . . . . . . .    
151	. G . . . . . . . . . .    
152	. A . . . . . . . . . .    
153	. C . . . . . . . . . .    
154	. T . . . . . . . . . .    
155	. A . . . . . . . . . .    
156	G G . . . . . . . . . .    
157	G G . . . . . . . . . .    
158	T T . . . . . . . . . .    
159	T T . . . . . . . . . .    
160	T T . . . . . . . . . .    
161	A A . . . . . . . . . .    
162	T T T . . . . . . . . .    
163	T T T . . . . . . . . .    
164	T T T . . . . . . . . .    
165	A A A . . . . . . . . .    
166	A A A . . . . . . . . .    
167	G G G . . . . . . . . .    
168	A A A . . . . . . . . .    
169	G G G . . . . . . . . .    
170	A A A . . . . . . . . .    
171	G G G . . . . . . . . .    
172	G G G . . . . . . . . .    
173	G G G . . . . . . . . .    
174	T T T . . . . . . . . .    
175	A A A . . . . . . . . .    
176	A A A . . . . . . . . .    
177	C C C . . . . . . . . .    
178	T T T . . . . . . . . .    
179	G G G . . . . . . . . .    
180	T T T . . . . . . . . .    
181	A A A . . . . . . . . .    
182	C C C . . . . . . . . .    
183	T T T . . . . . . . . .    
184	A A A . . . . . . . . .    
185	C C C . . . . . . . . .    
186	A A A . . . . . . . . .    
187	T T T . . . . . . . . .    
188	G G G . . . . . . . . .    
189	G G G . . . . . . . . .    
190	G G G . . . . . . . . .    
191	T T T . . . . . . . . .    
192	G G G . . . . . . . . .    
193	A A A . . . . . . . . .    
194	T T T . . . . . . . . .    
195	C C C . . . C . . . . .    
196	T T T . . . T . . . . .    
197	A A A . . . A . . . . .    
198	C C C . . . C . . . . .    
199	T T T . . . T . . . . .    
200	G G G . . . G . . . . .    
201	A A A . . . A . . . . .    
202	C C C . . . C . . . . .    
203	T T T . . . T . . . . .    
204	C C C . . . C . . . . .    
205	C C C . . . C C . . . .    
206	C C C . . . C C . . . .    
207	A A A . . . A A . . . .    
208	G G G . . . G G . . . .    
209	T T T . . . T T . . . .    
210	A A A . . . A A . . . .    
211	C C C . . . C C . . . .    
212	T T T . . . T T . . . .    
213	T T T . . . T T . . . .    
214	T T T . . . T T . . . .    
215	G G G . . . G G . . . .    
216	A A A . . . A A . . . .    
217	C C C . . . C C . . . .    
218	T T T . . . T T . . . .    
219	G G G . . . G G . . . .    
220	T T T . . . T T . . . .    
221	C C C . . . C C . . . .    
222	A A A . . . A A . . . .    
223	G G G . . . G G . . . .    
224	T T T . . . T T . . . .    
225	G G G . . . G G . . . .    
226	A A A . . . A A . . . .    
227	C C C . . . C C . . . .    
228	T T T . . . T T . . . .    
229	T T T . . . T T . . . .    
230	G G G . . . G G . . . .    
231	A A A . . . A A . . . .    
232	T T T . . . T T . . . .    
233	G G G . . . G G . . . .    
234	C C C . . . C C . . . .    
235	C C C . . . C C . . . .    
236	A A A . . . A A . . . .    
237	C C C . . . C C . . . .    
238	T T T . . . T T . . . .    
239	C C C . . . C C . . . .    
240	C C C . . . C C . . . .    
241	A A A . . . A A . . . .    
242	C C C . . . C C . . . .    
243	C C C . . . C C . . . .    
244	A A A . . . A A . . . .    
245	C C C . . . C C . . . .    
246	T T T . . . T T . . . .    
247	C C C . . . C C . . . .    
248	A A A . . . A A . . . .    
249	A A A . . . A A . . . .    
250	T T T . . . T T . . . .    
251	G G G . . . G G . . . .    
252	A A A . . . A A . . . .    
253	C C C . . . C C . . . .    
254	G G G . . . G G . . . .    
255	T T T . . . T T . . . .    
256	G G G . . . G G . . . .    
257	G G G . . . G G . . . .    
258	G G G . . . G G . . . .    
259	C C C . . . C C . . . .    
260	T T T . . . T T . . . .    
261	G G G . . . G G . . . .    
262	T T T . . . T T . . . .    
263	A A A . . . A A . . . .    
264	G G G . . . G G . . . .    
265	T T T . . . T T . . . .    
266	G G G . . . G G . . . .    
267	G G G . . . G G . . . .    
268	T T T . . . T T . . . .    
269	A A A . . . A A . . . .    
270	G G G . . . G G . . . .    
271	G G G . . . G G . . . .    
272	T T T . . . T T . . . .    
273	G G G . . . G G . . . .    
274	G G G . . . G G . . . .    
275	A A A . . . A A . . . .    
276	G G G . . . G G . . . .    
277	C C C . . . C C . . . .    
278	G G G . . . G G . . . .    
279	G G G . . . G G . . . .    
280	T T T . . . T T . . . .    
281	A A A . . . A A . . . .    
282	G G G . . . G G . . . .    
283	G G G . . . G G . . . .    
284	C C C . . . C C . . . .    
285	G G G . . . G G . . . .    
286	G G G . . . G G . . . .    
287	A A A . . . A A . . . .    
288	G G G . . . G G . . . .    
289	C C C . . . C C . . . .    
290	G G G . . . G G . . . .    
291	T T T . . . T T . . . .    
292	C C C . . . C C . . . .    
293	T T T . . . T T . . . .    
294	G G G . . . G G . . . .    
295	C C C . . . C C . . . .    
296	T T T . . . T T . . . .    
297	T T T . . . T T . . . .    
298	C C C . . . C C . . . .    
299	T T T . . . T T . . . .    
300	A A A . . . A A . . . .    
301	T T T . . . T T . . . .    
302	A A A . . . A A . . . .    
303	A A A . . . A A . . . .    
304	A A A . . . A A . . . .    
305	A A A . . . A A . . . .    
306	G G G . . . G G . . . .    
307	A A A . . . A A . . . .    
308	A A A . . . A A . . . .    
309	C C C . . . C C . . . .    
310	A A A . . . A A . . . .    
311	C C C . . . C C . . . .    
312	A A A . . . A A . . . .    
313	G G G . . . G G . . . .    
314	A A A . . . A A . . . .    
315	A A A . . . A A . . . .    
316	A A A . . . A A . . . .    
317	G G G . . . G G . . . .    
318	A A A . . . A A . . . .    
319	A A A . . . A A . . . .    
320	A A A . . . A A . . . .    
321	C C C . . . C C . . . .    
322	T T T . . . T T . . . .    
323	C C C . . . C C . . . .    
324	T T T . . . T T . . . .    
325	G G G . . . G G . . . .    
326	G G G . . . G G . . . .    
327	C C C . . . C C . . . .    
328	A A A . . . A A . . . .    
329	T T T . . . T T . . . .    
330	T T T . . . T T . . . .    
331	T T T . . . T T . . . .    
332	G G G . . . G G . . . .    
333	T T T . . . T T . . . .    
334	T T T . . . T T . . . .    
335	T T T . . . T T . . . .    
336	G G G . . . G G G . . .    
337	A A A . . . A A A . . .    
338	T T T . . . T T T . . .    
339	T T T . . . T T T . . .    
340	C C C . . . C C C . . .    
341	A A A . . . A A A . . .    
342	A A A . . . A A A . . .    
343	T T T . . . T T T . . .    
344	C C C . . . C C C . . .    
345	A A A . . . A A A . . .    
346	C C C . . . C C C . . .    
347	T T T . . . T T T . . .    
348	T T T . . . T T T . . .    
349	G G G . . . G G G . . .    
350	A A A . . . A A A . . .    
351	G G G . . . G G G . . .    
352	A A A . . . A A A . . .    
353	T T T . . . T T T . . .    
354	T T T . . . T T T . . .    
355	G G G . . . G G G . . .    
356	G G G . . . G G G . . .    
357	T T T . . . T T T . . .    
358	G G G . . . G G G . . .    
359	T T T . . . T T T . . .    
360	T T T . . . T T T . . .    
361	A A A . . . A A A . . .    
362	A A A . . . A A A . . .    
363	T T T . . . T T T . . .    
364	A A A . . . A A A . . .    
365	A A A . . . A A A . . .    
366	T T T . . . T T T . . .    
367	A A A . . . A A A . . .    
368	T T T . . . T T T . . .    
369	T T T . . . T T T . . .    
370	T T T . . . T T T . . .    
371	G G G . . . G G G . . .    
372	T T T . . . T T T . . .    
373	T T T . . . T T T . . T    
374	T T T . . . T T T . . T    
375	A A A . . . A A A . . A    
376	A A A . . . A A A . . A    
377	T T T . . . T T T . . T    
378	G G G . . . G G G . . G    
379	T T T . . . T T T . . T    
380	A A A . . . A A A . . A    
381	A A A . . . A A A . . A    
382	A A A . . . A A A . . A    
383	G G G . . . G G G . . G    
384	G G G . . . G G G . . G    
385	T T T . . . T T T . . T    
386	A A A . . . A A A . . A    
387	T T T . . . T T T . . T    
388	G G G . . . G G G . . G    
389	A A A . . . A A A . . A    
390	T T T . . . T T T . . T    
391	G G G . . . G G G . . G    
392	G G G . . . G G G . . G    
393	C C C . . . C C C . . C    
394	C C C . . . C C C . . C    
395	A A A . . . A A A . . A    
396	T T T . . . T T T . . T    
397	A A A . . . A A A . . A    
398	T T T . . . T T T . . T    
399	A A A . . . A A A . A A    
400	A A A . . . A A A . A A    
401	C C C . . . C C C . C C    
402	T T T . . . T T T . T T    
403	G G G . . . G G G . G G    
404	A A A . . . A A A . A A    
405	T T T . . . T T T . T T    
406	T T T . . . T T T . T T    
407	T T T . . . T T T . T T    
408	G G G . . . G G G . G G    
409	A A A . . . A A A . A A    
410	G G G . . . G G G . G G    
411	C C C . . . C C C . C C    
412	T T T . . . T T T . T T    
413	G G G G G . G G G . G G    
414	T T T T T . T T T . T T    
415	G G G G G . G G G . G G    
416	T T T T T . T T T . T T    
417	C C C C C . C C C . C C    
418	T T T T T . T T T . T T    
419	C C C C C . C C C . C C    
420	T T T T T . T T T . T T    
421	G G G G G . G G G . G G    
422	T T T T T . T T T . T T    
423	T T T T T . T T T . T T    
424	G G G G G . G G G . G G    
425	G G G G G . G G G . G G    
426	G G G G G . G G G . G G    
427	T T T T T . T T T . T T    
428	G G G G G . G G G . G G    
429	G G G G G . G G G . G G    
430	A A A A A . A A A . A A    
431	A A A A A . A A A . A A    
432	A A A A A . A A A . A A    
433	A A A A A . A A A . A A    
434	G G G G G . G G G . G G    
435	G G G G G . G G G . G G    
436	T T T T T . T T T . T T    
437	A C C C C . A C C . A C    
438	A A A A A . A A A . A A    
439	T T T T T . T T T . T T    
440	A A A A A . A A A . A A    
441	T T T T T . T T T . T T    
442	G G G G G . G G G . G G    
443	T T T T T . T T T . T T    
444	T T T T T . T T T . T T    
445	T T T T T . T T T . T T    
446	T T T T T . T T T . T T    
447	G G G G G . G G G . G G    
448	A A A A A . A A A . A A    
449	G G G G G . G G G . G G    
450	T T T T T . T T T T T T    
451	G G G G G . G G G G G G    
452	T T T T T . T T T T T T    
453	T T T T T . T T T T T T    
454	G G G G G . G G G G G G    
455	G G G G G . G G G G G G    
456	T T T T T . T T T T T T    
457	T T T T T . T T T T T T    
458	T T T T T . T T T T T A    
459	G G G G G . G G G G G G    
460	T T T T T . T T T T T T    
461	G G G G G . G G G G G G    
462	T T T T T . T T T T T T    
463	T T T T T . T T T T T T    
464	T T T T T . T T T T T T    
465	T T T T T . T T T T T T    
466	T T T T T . T T T T T T    
467	G G G G G . G G G G G G    
468	A A A A A . A A A A A A    
469	T T T T T . T T T T T T    
470	G G G G G . G G G G G G    
471	T T T T T . T T T T T T    
472	G G G G G . G G G G G G    
473	C C C C C . C C C C C C    
474	C C C C C . C C C C C C    
475	C C C C C . C C C C C C    
476	T T T T T . T T T T T T    
477	G G G G G G G G G G G G    
478	G G G G G G G G G G G G    
479	T T T T T T T T T T T T    
480	C C C C C C C C C C C C    
481	C C C C C C C C C C C C    
482	A A A A A A A A A A A A    
483	A A A A A A A A A A A A    
484	T T T T T T T T T T T T    
485	G G G G G G G G G G G G    
486	G G G G G G G G G G G G    
487	T T T T T T T T T T T T    
488	T T T T T T T T T T T T    
489	T T T T T T T T T T T T    
490	C C C C C C C C C C C C    
491	C C C C C C C C C C C C    
492	A A A A A A A A A A A A    
493	A A A A A A A A A A A A    
494	C C C C C C C C C C C C    
495	A A A A A A A A A A A A    
496	A A A A A A A A A A A A    
497	A A A A A A A A A A A A    
498	C C C C C C C C C C C C    
499	T T T T T T T T T T T T    
500	G G G G G G G G G G G G    
501	T T T T T T T T T T T T    
502	C C C C C C C C C C C C    
503	A A A A A A A A A A A A    
504	T T T T T T T T T T T T    
505	T T T T T T T T T T T T    
506	T T T T T T T T T T T T    
507	T T T T T T T T T T T T    
508	A A A A A A A A A A A A    
509	A A A A A A A A A A A A    
510	T T T T T T T T T T T T    
511	G G G G G G G G G G G G    
512	C C C C C C C C C C C C    
513	A A A A A A A A A A A A    
514	G G G G G G G G G G G G    
515	A A A A A A A A A A A A    
516	T T T T T T T T T T T T    
517	T T T T T T T T T T T T    
518	G G G G G G G G G G G G    
519	A A A A A A A A A A A A    
520	T T T T T T T T T T T T    
521	T T T T T T T T T T T T    
522	A A A A A A A A A A A A    
523	T T T T T T T T T T T T    
524	T T T T T T T T T T T T    
525	A A A A A A A A A A A A    
526	C C C C C C C C C C C C    
527	A A A A A A A A A A A A    
528	T T T T T T T T T T T T    
529	G G G G G G G G G G G G    
530	T T T T T T T T T T T T    
531	T T T T T T T T T T T T    
532	A A A A A A A A A A A A    
533	A A A A A A A A A A A A    
534	T T T T T T T T T T T T    
535	G G G G G G G G G G G G    
536	C C C C C C C C C C C C    
537	C C C C C C C C C C C C    
538	A A A A A A A A A A A A    
539	C C C C C C C C C C C C    
540	A A A A A A A A A A A A    
541	A A A A A A A A A A A A    
542	A A A A A A A A A A A A    
543	C C C C C C C C C C C C    
544	A A A A A A A A A A A A    
545	T T T T T T T T T T T T    
546	G G G G G G G G G G G G    
547	G G G G G G G G G G G G    
548	C C C C C C C C C C C C    
549	A A A A A A A A A A A A    
550	T T T T T T T T T T T T    
551	T T T T T T T T T T T T    
552	T T T T T T T T T T T T    
553	G G G G G G G G G G G G    
554	T T T T T T T T T T T T    
555	T T T T T T T T T T T T    
556	T T T T T T T T T T T T    
557	C C C C C C C C C C C T    
558	T T T T T T T T T T T T    
559	G G G G G G G G G G G G    
560	A A A A A A A A A A A A    
561	A A A A A A A A A A A A    
562	A A A A A A A A A A A A    
563	A A A A A A A A A A A A    
564	T T T T T T T T T T T T    
565	G G G G G G G G G G G G    
566	G G G G G G G G G G G G    
567	C C C C C C C C C C C C    
568	A A A A A A A A A A A A    
569	T T T T T T T T T T T T    
570	T T T T T T T T T T T T    
571	G G G G G G G G G G G G    
572	T T T T T T T T T T T T    
573	T T T T T T T T T T T T    
574	T T T T T T T T T T T T    
575	G G G G G G G G G G G G    
576	T T T T T T T T T T T T    
577	A A A A A A A A A A A A    
578	C C C C C C C C C C C C    
579	T T T T T T T T T T T T    
580	A A A A A A A A A A A A    
581	A A A A A A A A A A A A    
582	A A A A A A A A A A A A    
583	A A A A A A A A A A A A    
584	G G G G G G G G G G G G    
585	G G G G G G G G G G G G    
586	C C C C C C C C C C C C    
587	A A A A A A A A A A A A    
588	T T T T T T T T T T T T    
589	T T T T T T T T T T T T    
590	T T T T T T T T T T T T    
591	T T T T T T T T T T T T    
592	G G G G G G G G G G G G    
593	A A A A A A A A A A A A    
594	C C C C C C C C C C C C    
595	A A A A A A A A A A A A    
596	C C C C C C C C C C C C    
597	T T T T T T T T T T T T    
598	G G G G G G G G G G G G    
599	A A A A A A A A A A A A    
600	A A A A A A A A A A A A    
601	A A A A A A A A A A A A    
602	G G G G G G G G G G G G    
603	G G G G G G G G G G G G    
604	T T T T T T T T T T T T    
605	T T T T T T T T T T T T    
606	A A A A A A A A A A A A    
607	T T T T T T T T T T T T    
608	T T T T T T T T T T T T    
609	T T T T T T T T T T T T    
610	G G G G G G G G G G G G    
611	G G G G G G G G G G G G    
612	A A A A A A A A A A A A    
613	C C C C C C C C C C C C    
614	A A A A A A A A A A A A    
615	T T T T T T T T T T T T    
616	A A A A A A A A A A A A    
617	A A A A A A A A A A A A    
618	T T T T T T T T T T T T    
619	T T T T T T T T T T T T    
620	G G G G G G G G G G G G    
621	T T T T T T T T T T T T    
622	A A A A A A A A A A A A    
623	C C C C C C C C C C C C    
624	A A A A A A A A A A A A    
625	C C C C C C C C C C C C    
626	A A A A A A A A A A A A    
627	C C C C C C C C C C C C    
628	T T T T T T T T T T T T    
629	A A A A A A A A A A A A    
630	C C C C C C C C C C C C    
631	A A A A A A A A A A A A    
632	A A A A A A A A A A A A    
633	A A A A A A A A A A A A    
634	T T T T T T T T T T T T    
635	A A A A A A A A A A A A    
636	T T T T T T T T T T T T    
637	T T T T T T T T T T T T    
638	A A A A A A A A A A A A    
639	C C C C C C C C C C C C    
640	T T T T T T T T T T T T    
641	C C C C C C C C C C C C    
642	T T T T T T T T T T T T    
643	T T T T T T T T T T T T    
644	T T T T T T T T T T T T    
645	C C C C C C C C C C C C    
646	A A A A A A A A A A A A    
647	A A A A A A A A A A A A    
648	A A A A A A A A A A A A    
649	T T T T T T T T T T T T    
650	A A A A A A A A A A A A    
651	T T T T T T T T T T T T    
652	T T T T T T T T T T T T    
653	G G G G G G G G G G G G    
654	T T T T T T T T T T T T    
655	T T T T T T T T T T T T    
656	G G G G G G G G G G G G    
657	C C C C C C C C C C C C    
658	A A A A A A A A A A A A    
659	A A A A A A A A A A A A    
660	T T T T T T T T T T T T    
661	C C C C C C C C C C C C    
662	T T T T T T T T T T T T    
663	T T T T T T T T T T T T    
664	C C C C C C C C C C C C    
665	C C C C C C C C C C C C    
666	A A A A A A A A A A A A    
667	A A A A A A A A A A A A    
668	C C C C C C C C C C C C    
669	A A A A A A A A A A A A    
670	G G G G G G G G G G G G    
671	G G G G G G G G G G G G    
672	T T T T T T T T T T T T    
673	C C C C C C C C C C C C    
674	T T T T T T T T T T T T    
675	A A A A A A A A A A A A    
676	T T T T T T T T T T T T    
677	T T T T T T T T T T T T    
678	T T T T T T T T T T T T    
679	T T T T T T T T T T T T    
680	T T T T T T T T T T T T    
681	G G G G G G G G G G G G    
682	G G G G G G G G G G G G    
683	C C C C C C C C C C C C    
684	A A A A A A A A A A A A    
685	A A A A A A A A A A A A    
686	A A A A A A A A A A A A    
687	A A A A A A A A A A A A    
688	A A A A A A A A A A A A    
689	A A A A A A A A A A A A    
690	A A A A A A A A A A A A    
691	- A A A A A A A A A - A    
692	- A A - - A A A A A - A  4	 indel  
693	T T T T T T T T T T T T    
694	G G G G G G G G G G G G    
695	T T T T T T T T T T T T    
696	T T T T T T T T T T T T    
697	T T T T T T T T T T T T    
698	G G G G G G G G G G G G    
699	G G G G G G G G G G G G    
700	G G G G G G G G G G G G    
701	A A A A A A A A A A A A    
702	G G G G G G G G G G G G    
703	A A A A A A A A A A A A    
704	T T T T T T T T T T T T    
705	T T T T T T T T T T T T    
706	T T T T T T T T T T T T    
707	A A A A A A A A A A A A    
708	C C C C C C C C C C C C    
709	T T T T T T T T T T T T    
710	A A A A A A A A A A A A    
711	T T T T T T T T T T T T    
712	T T T T T T T T T T T T    
713	T T T T T T T T T T T T    
714	A A A A A A A A A A A A    
715	A A A A A A A A A A A A    
716	T T T T T T T T T T T T    
717	G G G G G G G G G G G G    
718	G G G G G G G G G G G G    
719	C C C C C C C C C C C C    
720	A A A A A A A A A A A A    
721	G G G G G G G G G G G G    
722	A A A A A A A A A A A A    
723	A A A A A A A A A A A A    
724	A A A A A A A A A A A A    
725	A A A A A A A A A A A A    
726	T T T T T T T T T T T T    
727	A A A A A A A A A A A A    
728	T T T T T T T T T T T T    
729	C C C C C C C C C C C C    
730	T T T T T T T T T T T T    
731	T T T T T T T T T T T T    
732	C C C C C C C C C C C C    
733	T T T T T T T T T T T T    
734	A A A A A A A A A A A A    
735	T T T T T T T T T T T T    
736	A A A A A A A A A A A A    
737	C C C C C C C C C C C C    
738	T T T T T T T T T T T T    
739	A A A A A A A A A A A A    
740	G G G G G G G G G G G G    
741	T T T T T T T T T T T T    
742	C C C C C C C C C C C C    
743	T T T T T T T T T T T T    
744	C C C C C C C C C C C C    
745	A A A A A A A A A A A A    
746	A A A A A A A A A A A A    
747	T T T T T T T T T T T T    
748	A A A A A A A A A A A A    
749	T T T T T T T T T T T T    
750	A A A A A A A A A A A A    
751	C C C C C C C C C C C C    
752	A A A A A A A A A A A A    
753	A A A A A A A A A A A A    
754	G G G G G G G G G G G G    
755	G G G G G G G G G G G G    
756	C C C C C C C C C C C C    
757	T T T T T T T T T T T T    
758	T T T T T T T T T T T T    
759	A A A A A A A A A A A A    
760	T T T T T T T T T T T T    
761	T T T T T T T T T T T T    
762	T T T T T T T T T T T T    
763	C C C C C C C C C C C C    
764	A A A A A A A A A A A A    
765	A A A A A A A A A A A A    
766	T T T T T T T T T T T T    
767	G G G G G G G G G G G G    
768	C C C C C C C C C C C C    
769	C C C C C C C C C C C C    
770	T T T T T T T T T T T T    
771	A A A A A A A A A A A A    
772	T T T T T T T T T T T T    
773	T T T T T T T T T T T T    
774	T T T T T T T T T T T T    
775	G G G G G G G G G G G G    
776	G G G G G G G G G G G G    
777	G G G G G G G G G G G G    
778	T T T T T T T T T T T T    
779	T T T T T T T T T T T T    
780	G G G G G G G G G G G G    
781	C C C C C C C C C C C C    
782	A A A A A A A A A A A A    
783	G G G G G G G G G G G G    
784	T T T T T T T T T T T T    
785	A A A A A A A A A A A A    
786	T T T T T T T T T T T T    
787	T T T T T T T T T T T T    
788	A A A A A A A A A A A A    
789	A A A A A A A A A A A A    
790	G G G G G G G G G G G G    
791	C C C C C C C C C C C C    
792	C C C C C C C C C C C C    
793	A A A A A A A A A A A A    
794	T T T T T T T T T T T T    
795	C C C C C C C C C C C C    
796	T T T T T T T T T T T T    
797	T T T T T T T T T T T T    
798	G G G G G G G G G G G G    
799	T T T T T T T T T T T T    
800	G G G G G G G G G G G G    
801	A A A A A A A A A A A A    
802	A A A A A A A A A A A A    
803	T T T T T T T T T T T T    
804	G G G G G G G G G G G G    
805	T T T T T T T T T T T T    
806	A A A A A A A A A A A A    
807	T T T T T T T T T T T T    
808	T T T T T T T T T T T T    
809	T T T T T T T T T T T T    
810	C C C C C C C C C C C C    
811	C C C C C C C C C C C C    
812	A A A A A A A A A A A A    
813	T T T T T T T T T T T T    
814	G G G G G G G G G G G G    
815	A A A A A A A A A A A A    
816	G G G G G G G G G G G G    
817	A A A A A A A A A A A A    
818	T T T T T T T T T T T T    
819	G G G G G G G G G G G G    
820	. T T T T T T T T T T T    
821	. T T T T T T T T T T T    
822	. A A A A A A A A A A A    
823	. G G G G G G G G G G G    
824	. A A A A A A A A A A A    
825	. A A A A A A A A A A A    
826	. G G G G G G G G G G G    
827	. A A A A A A A A A A A    
828	. G G G G G G G G G G G    
829	. - - - - N - - - - - -    
830	. C C C C C C C C C C C    
831	. A A A A A A A A A A A    
832	. G G G G G G G G G G G    
833	. T T T T T T T T T T T    
834	. G G G G G G G G G G G    
835	. T T T T T T T T T T T    
836	. T T T T - - T T - - T  4	 indel  
837	. T T T T - - T T - - T  4	 indel  
838	. G G G G G G G G G G G    
839	. T T T T T T T T T T T    
840	. A A A A A A A A A A A    
841	. T T T T T T T T T T T    
842	. G G G G G G G G G G G    
843	. T T T T T T T T T T T    
844	. A A A A A A A A A A A    
845	. T T T T T T T T T T T    
846	. T T T T T T T T T T T    
847	. T T T T T T T T T T T    
848	. T T T T T T T T T T T    
849	. A A A A A A A A A A A    
850	. A A A A A A A A A A A    
851	. A A A A A A A A A A A    
852	. G G G G G G G G G G G    
853	. G G G G G G G G G G G    
854	. A A A A A A A A A A A    
855	. C C C C C C C C C C C    
856	. T T T T T T T T T T T    
857	. G G G G G G G G G G G    
858	. T T T T T T T T T T T    
859	. T T T T T T T T T T T    
860	. T T T T T T T T T T T    
861	. T G G G G G G G G G G    
862	. . A A A A A A A A A A    
863	. . T T T T T T T T T T    
864	. . G G G G G G G G G G    
865	. . G G G G G G G G G G    
866	. . A A A A A A A A A A    
867	. . A A A A A A A A A A    
868	. . A A A A A A A A A A    
869	. . C C C C C C C C C C    
870	. . C C C C C C C C C C    
871	. . G G G G G G G G G G    
872	. . T T T T T T T T T T    
873	. . A A A A A A A A A A    
874	. . G G G G G G G G G G    
875	. . A A A A A A A A A A    
876	. . A A A A A A A A A A    
877	. . G G G G G G G G G G    
878	. . A A A A A A A A A A    
879	. . T T T T T T T T T T    
880	. . G G G G G G G G G G    
881	. . T T T T T T T T T T    
882	. . C C C C C C C C C C    
883	. . C C C C C C C C C C    
884	. . T T T T T T T T T T    
885	. . T T T T T T T T T T    
886	. . A A A A A A A A A A    
887	. . A A A A A A A A A A    
888	. . A A A A A A A A A A    
889	. . C C C C C C C C C C    
890	. . A A A A A A A A A A    
891	. . G G G G G G G G G G    
892	. . T T T T T T T T T T    
893	. . G G G G G G G G G G    
894	. . T T T T T T T T T T    
895	. . A A A A A A A A A A    
896	. . A A A A A A A A A A    
897	. . T T T T T T T T T T    
898	. . A A A A A A A A A A    
899	. . A A A A A A A A A A    
900	. . A A A A A A A A A A    
901	. . A A A A A A A A A A    
902	. . T T T T T T T T T T    
903	. . A A A A A A A A A A    
904	. . T T T T T T T T T T    
905	. . T T T T T T T T T T    
906	. . C C C C C C C C C C    
907	. . C C C C C C C C C C    
908	. . T T T T T T T T T T    
909	. . G G G G G G G G G G    
910	. . C C C C C C C C C C    
911	. . T T T T T T T T T T    
912	. . A A A A A A A A A A    
913	. . G G G G G G G G G G    
914	. . A A A A A A A A A A    
915	. . T T T T T T T T T T    
916	. . G G G G G G G G G G    
917	. . A A A A A A A A A A    
918	. . C C C C C C C C C C    
919	. . A A A A A A A A A A    
920	. . C C C C C C C C C C    
921	. . C C C C C C C C C C    
922	. . A A A A A A A A A A    
923	. . A A A A A A A A A A    
924	. . C C C C C C C C C C    
925	. . T T T T T T T T T T    
926	. . G G G G G G G G G G    
927	. . T T T T T T T T T T    
928	. . . . . . A A A A A A    
929	. . . . . . A A A A A A    
930	. . . . . . A A A A A A    
931	. . . . . . A A A A A A    
932	. . . . . . A A A A A A    
933	. . . . . . G G G G G G    
934	. . . . . . C C C C C C    
935	. . . . . . C C C C C C    
936	. . . . . . T T T T T T    
937	. . . . . . C C C C C C    
938	. . . . . . G G G G G G    
939	. . . . . . G G G G G G    
940	. . . . . . C C C C C C    
941	. . . . . . A A A A A A    
942	. . . . . . T T T T T T    
943	. . . . . . A A A A A A    
944	. . . . . . A A A A A A    
945	. . . . . . A A A A A A    
946	. . . . . . T T T T T T    
947	. . . . . . A A A A A A    
948	. . . . . . T T T T T T    
949	. . . . . . G G G G G G    
950	. . . . . . T T T T T T    
951	. . . . . . G G G G G G    
952	. . . . . . C C C C C C    
953	. . . . . . G G G G G G    
954	. . . . . . T T T T T T    
955	. . . . . . T T T T T T    
956	. . . . . . G G G G G G    
957	. . . . . . A A A A A A    
958	. . . . . . C C C C C C    
959	. . . . . . A A A A A A    
960	. . . . . . T T T T T T    
961	. . . . . . T T T T T T    
962	. . . . . . T T T T T T    
963	. . . . . . T T T T T T    
964	. . . . . . C C C C C C    
965	. . . . . . A A A A A A    
966	. . . . . . C C C C C C    
967	. . . . . . A A A A A A    
968	. . . . . . T T T T T T    
969	. . . . . . G T G G G G    
970	. . . . . . A . A A A A    
971	. . . . . . T . T T T T    
972	. . . . . . A . A A A A    
973	. . . . . . T . T T T T    
974	. . . . . . G . G G G G    
975	. . . . . . C . C C C C    
976	. . . . . . T . T T T T    
977	. . . . . . G . G G G G    
978	. . . . . . T . T T T T    
979	. . . . . . A . A A A A    
980	. . . . . . C . C C C C    
981	. . . . . . A . A A A A    
982	. . . . . . T . T T T T    
983	. . . . . . G . G G G G    
984	. . . . . . C . C C C C    
985	. . . . . . A . A A A A    
986	. . . . . . C . C C C C    
987	. . . . . . T . T T T T    
988	. . . . . . T . T T T T    
989	. . . . . . T . T T T T    
990	. . . . . . G . G G G G    
991	. . . . . . A . G G G G    
992	. . . . . . . . G G G G    
993	. . . . . . . . A A A A    
994	. . . . . . . . C C C C    
995	. . . . . . . . T T T T    
996	. . . . . . . . A A A A    
997	. . . . . . . . T T T T    
998	. . . . . . . . A A A A    
999	. . . . . . . . C C C C    
1000	. . . . . . . . T T T T    
1001	. . . . . . . . T T T T    
1002	. . . . . . . . C C C C    
1003	. . . . . . . . C C C C    
1004	. . . . . . . . G G G G    
1005	. . . . . . . . C C C C    
1006	. . . . . . . . T T T T    
1007	. . . . . . . . C C C C    
1008	. . . . . . . . C C C C    
1009	. . . . . . . . A A A A    
1010	. . . . . . . . T T T T    
1011	. . . . . . . . A A A A    
1012	. . . . . . . . T T T T    
1013	. . . . . . . . G G G G    
1014	. . . . . . . . A A A A    
1015	. . . . . . . . C C C C    
1016	. . . . . . . . A A A A    
1017	. . . . . . . . A A A A    
1018	. . . . . . . . A A A A    
1019	. . . . . . . . T T T T    
1020	. . . . . . . . G G G G    
1021	. . . . . . . . A A A A    
1022	. . . . . . . . T T T T    
1023	. . . . . . . . T T T T    
1024	. . . . . . . . A A A A    
1025	. . . . . . . . T T T T    
1026	. . . . . . . . T T T T    
1027	. . . . . . . . C C C C    
1028	. . . . . . . . T T T T    
1029	. . . . . . . . G G G G    
1030	. . . . . . . . T T T T    
1031	. . . . . . . . G G G G    
1032	. . . . . . . . G G G G    
1033	. . . . . . . . G G G G    
1034	. . . . . . . . T T T T    
1035	. . . . . . . . T T T T    
1036	. . . . . . . . T T T T    
1037	. . . . . . . . C C C C    
1038	. . . . . . . . C C C C    
1039	. . . . . . . . A A A A    
1040	. . . . . . . . T T T T    
1041	. . . . . . . . C C C C    
1042	. . . . . . . . C C C C    
1043	. . . . . . . . T T T T    
1044	. . . . . . . . T T T T    
1045	. . . . . . . . G G G G    
1046	. . . . . . . . C C C G    
1047	. . . . . . . . A A A .    
1048	. . . . . . . . A A A .    
1049	. . . . . . . . T T T .    
1050	. . . . . . . . A A A .    
1051	. . . . . . . . A A A .    
1052	. . . . . . . . A A A .    
1053	. . . . . . . . C C C .    
1054	. . . . . . . . A A A .    
1055	. . . . . . . . G G G .    
1056	. . . . . . . . T T T .    
1057	. . . . . . . . T T T .    
1058	. . . . . . . . G G G .    
1059	. . . . . . . . A A A .    
1060	. . . . . . . . C C C .    
1061	. . . . . . . . T T T .    
1062	. . . . . . . . A . A .    
1063	. . . . . . . . A . A .    
1064	. . . . . . . . A . A .    
1065	. . . . . . . . C . A .    
1066	. . . . . . . . T . . .    
1067	. . . . . . . . G . . .    
1068	. . . . . . . . G . . .
```
